# Metal accumulation in fish species of a vast hydrographic network in the Moyen-Ogooué and Haut-Ogooué Provinces of Gabon: Implications for human health

**DOI:** 10.1016/j.toxrep.2024.101842

**Published:** 2024-11-28

**Authors:** Clency Mikala Okouyi, Michel Mathurin Kamdem, Patricks Voua Otomo, Gaël Darren Maganga

**Affiliations:** a Department of Zootechnology, University of Science and Technology of Masuku, P.O Box 941, Franceville, Gabon; b Department of Zoology and Entomology, University of the Free State, Private Bag x13, Phuthaditjhaba 9866, South Africa; c Department of Environmental Engineering, University of N’Zérékoré, P.O Box 50, N’Zérékoré, Guinea; d Centre for Global Change, University of the Free State, Private Bag x13, Phuthaditjhaba 9866, South Africa; e Franceville Interdisciplinary Medical Research Center (CIRMF), P.O Box 769, Franceville, Gabon

**Keywords:** Heavy metals, Mercury, Aluminium, Iron, High concentration, Lake fish species, High carcinogenic risk, Gabon

## Abstract

With an obsolete livestock sector, Gabon relies on its huge hydrographic network rich in fish to supply its populations with animal proteins. This study aimed to conduct metal analyses in four fish species (*Oreochromis niloticus, Clarias gariepinus, Chrysichthys nigrodigitatus, Polydactylus quadrifilis*) frequently consumed by human populations in the Moyen-Ogooué and Haut-Ogooué Provinces of Gabon and infer the potential human health risks for those populations who rely on these freshwater produces as a source of proteins. Fish were sampled from Ezanga, Oguemoué, Onangué, Nguenè (Moyen-Ogooué) and Grand Poubara (Haut-Ogooué) Lakes during the high flow period (HF) and low flow period (LF) from 2021 to 2022, and analysed for seven heavy metals (HMs) using Inductively Coupled Plasma Optical Emission Spectroscopy (ICP-OES) techniques. Throughout the flow periods, and regardless of the lake and fish species, Fe was found to have the highest concentration, followed by Al > Mn > Hg > Pb> As> Cd. The relatively high concentration of Hg was recorded in the muscle tissues of *C. gariepinus* (6.65 mg. kg^−1^) sampled during the LF period at Grand Poubara. The concentrations of Hg found in the muscle of all fish species also exceeded the maximum acceptable limits set by the American Environmental Protection Agency. The concentrations of Fe in *C. gariepinus* (Grand Poubara, LF) and *O. niloticus* (Onangué, LF), and those of Al in *O. niloticus* (Nguenè, HF), *P. quadrifilis* (Onangué, HF) and *C. nigrodigitatus* (Oguemoué, LF) were amongst the highest concentrations ever reported on the African continent. Health risk assessments indicated a heightened risk of cancer for local populations consuming the fish species from all the lakes investigated. There is a need to implement an increased surveillance programme at national level in order to raise awareness and improve the management of fishery resources while preserving the environment and the health of local populations that rely upon these resources for their subsistence.

## Highlights

• First record of HMs in four fish species Nile tilapia, walking catfish, bagrid catfish and giant African threadfin found in hydrographic network of Gabon.
• Concentrations of HMs in muscle tissues depend upon fish species and seasonal water flow levels.
• All the five lakes in the Moyen-Ogooué and Haut-Ogooué Provinces are polluted by HMs originating mainly from anthropogenic activities.
• Fe and Al bioaccumulation in all the four fish species are amongst the highest ever reported on the African continent.
• A heightened risk of cancer for local populations consuming the fish species from all the lakes investigated.

## 1 Introduction

Fishing is one of the oldest activities practised by humans. Indeed, long before the advent of agriculture and livestock farming, fishing was done in small water bodies such as ponds and lagoons and much later in big water bodies including lakes, rivers and seas [1]. In the modern world, fishing plays an increasingly important economic and social role as it contributes substantially to food security and job creation. By 2010, fishing was a source of income for more than one hundred million people worldwide [1]. While in terms of proportion marine fishing (of which fish account for 85 % of the total production) remains predominant (78.8 million tonnes per year), that of inland fishing (11.5 million tonnes per year) should not be overlooked [2]. In Africa, 12.3 million people depend on inland fishing as their main source of income and nearly 200 million Africans have fish as their main source of protein [3], [4], with an annual production of more than 2.8 million tonnes [4].

Gabon is among the five largest consumers of fish in Africa, with an annual consumption estimated between 30 and 34.5 kg per capita. In 2017, the Gabonese fishing sector mobilised more than 29,700 people for an annual production around 29,000 tonnes [5]. In the country, inland fishing contributes for 37 % of the total fish production [5]. The Southern Lakes area, located in the Moyen-Ogooué Province, concentrates about 44 % of the inland production, the rest being distributed in other areas such as the Ogooué-Ivindo, Ogooué-Lolo, Nyanga and Haut-Ogooué Provinces. Fishing activities, however, are threatened by intense anthropogenic activities around and/or in the rivers and lakes. These activities include mining (gold, manganese), hydrocarbon extractions (gas and oil), wood exploitation, processing, transport, chemical and food industries. Environmental contaminants such as heavy metals (HMs) are known, for some, to be abundant elements of the earth’s crust and for others to be ubiquitous anthropogenic pollutants in aquatic ecosystems [6], [7]. Because of their long-term persistence, HMs are known to bioaccumulate in the tissues of aquatic organisms such as fish [8]. HMs are known to have deleterious effects on humans and other life forms and as such monitoring their bioaccumulation is crucial for the assessment of potential toxicological risks [9], [10], [11]. Fish are at the higher level of the food chain in freshwater ecosystems and they are widely used to biologically monitor the degree of metal pollution from the environment they live in following various parameters such as species characteristics, exposure time, concentration of individual metals either in tissues and/or water samples [11], [12]. Human populations consuming fish with relatively large amounts of bioaccumulated HMs can be at risk of devastating and irreversible health effects, resulting in mutagenesis, carcinogenesis, neurological damage, DNA damage, immune system, neurological, and renal disorders [13], [14], [15].

The aims of the present study were to conduct metal analyses in four fish species (*Oreochromis niloticus, Clarias gariepinus, Chrysichthys nigrodigitatus, Polydactylus quadrifilis*) frequently consumed by human populations in the Moyen-Ogooué and Haut-Ogooué Provinces of Gabon and infer the potential human health risks for those populations who rely on these freshwater produces as a source of proteins.

## 2 Materials and methods

### 2.1 Study area

**Fig. 1:**
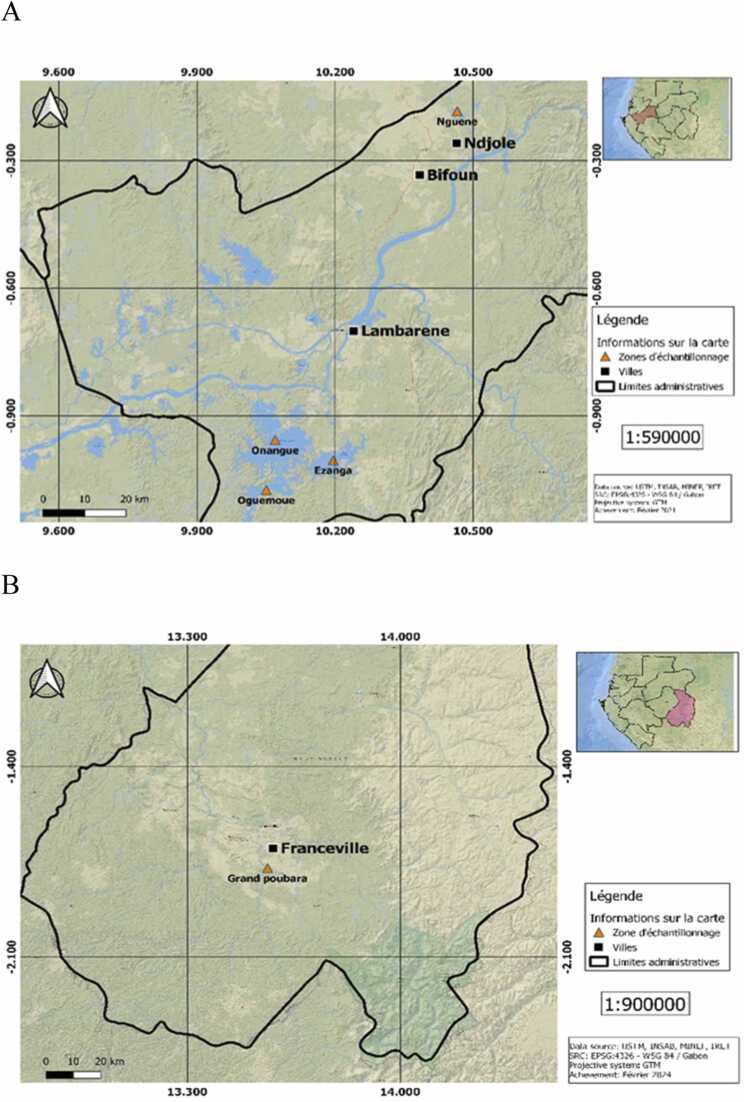
Map of the study area showing the fish sampling lakes in the A) Moyen-Ogooué and B) Haut-Ogooué Provinces of Gabon.

The study took place within the Moyen-Ogooué and the Haut-Ogooué Provinces of Gabon, which encompass, respectively, the Great Lakes Region and the Grand Poubara Dam. In the Moyen-Ogooué Province, fish sampling occurred in the Great Lakes Region including Ezanga Lake, Oguemoué Lake and Onangué Lake (Fig. 1A). An additional site, Nguenè Lake, located to the west of the region, is known for having fewer fishing activities [16]. The last sampling site was the Grand Poubara Dam (Fig. 1B), whose water reservoir is approximately 46 km^2^, and around which small lakes have formed, promoting the development of fishing activities. The climate of both these provinces is of an equatorial type, characterized by two rainy seasons and two dry seasons, with annual rainfall topping 1831 mm and an average annual temperature of approximately 28ºC [16]. The hydrographic network of the study area is relatively dense and includes the Ogooué River and several lakes which favour artisanal fishing, agriculture and agro-industry activities [17].

### 2.2 Fish sampling

Fish sampling was carried out during the dry and rainy seasons, which corresponded to the low and high river flow periods, respectively. Fish sampling from Ezanga, Oguemoué and Onangué Lakes took place during the last half of April (HF period) and in the first half of August 2021 (LF period) for the first campaign and during the same periods for the second campaign in 2022. For the Grand Poubara Dam, sampling was done during the first half of May (HF period) and the last half of August (LF period) for the two campaigns. A total of 651 fish including the species *Oreochromis niloticus* (388 individuals), *Chrysichthys nigrodigitatus* (102 individuals), *Clarias gariepinus* (101 individuals) and *Polydactylus quadrifilis* (60 individuals) were collected across the different sites (Table 1). Fish samples were identified using the identification keys provided by Mbega and Teugels [18].

**Table 1:** Number of fish species collected across the studied lakes.

Lakes	Water flow	Species				
		*O. niloticus*	*C. nigrodigitatus*	*P. quadrifilis*	*C. gariepinus*	Total
Oguemoué	HF	20	31	0	0	51
	LF	0	10	0	0	10
Ezanga	HF	30	0	0	0	30
	LF	20	18	11	0	49
Onangué	HF	0	0	14	0	14
	LF	30	30	22	0	82
Nguenè	HF	21	3	6	0	30
	LF	23	10	7	0	40
Grand Poubara	HF	138	0	0	67	205
	LF	106	0	0	34	140
		388	102	60	101	651

HF= high flow; LF = low flow

### 2.3 Preparation of samples and heavy metal analyses

The fish collected were scaled, gutted and dissected. From each individual, fish fillets were assembled from both sides of the animal, in order to obtain as much muscle as possible. The choice of fish fillets was guided by the fact that it is the part most frequently consumed by local populations. Each sample was weighed and placed in an oven at 80ºC for 48 h. The dried samples were mineralized in a microwave digester and then transferred to a flask containing 6 ml of 65 % nitric acid (HNO_3_) and 2 ml of 30 % hydrogen peroxide (H_2_O_2_). A blank was run with all batches. After digestion in the Milestone Ethos microwave, the samples were diluted with distilled water up to 50 ml, then run on the Inductively Coupled Plasma Optical Emission Spectroscopy (ICP-OES) using the standard program. The blank was subtracted from the results received. Metal analyses were conducted by Waterlab (Pty) Ltd in Pretoria, Gauteng Province, South Africa. Five measurements for all muscle samples were performed for heavy metals (HMs) analysis. Seven HMs were searched including aluminum (Al), arsenic (As), cadmium (Cd), iron (Fe), manganese (Mn), mercury (Hg) and lead (Pb). All metal concentrations were measured and expressed as mg.kg^−1^ dry weight (dw) in fish samples.

### 2.4 Heavy metal index for fish pollution assessment

To monitor the total contamination of HMs in fish species at the different sampling sites, the Metal Pollution Index (MPI) was calculated as the geometric mean of values for HMs in fish muscle following the equation [19]:

MPI (mg.kg^−1^ dw) = (MC_1_ × MC_2_ × MC_3_……MC_n_)^1/n^

MC_1_ is the concentration of metal 1 in the fish sample (mg.kg^−1^ dw), and n is the number of metals. The higher the index, the greater the likelihood of accumulating metals that may pose a risk to human health.

### 2.5 Human health risk assessment

#### 2.5.1 Estimated daily intake (DI) of metals

The average daily food intakes (DI) of groups of metals from the four species of fish were calculated as follows:

DI (mg.kg^−1^ day^−1^) = MC × DR / BW

Where MC is the average concentration of HMs in fish (mg·kg^−1^ dw), DR is the estimated daily consumption rate of fish (g/person/day), set at 82 g/person/day (that is 30 kg/person/year in Gabon) as reported by the Food and Agriculture Organization [5]. BW is the average body weight (kg) for adult people in Gabon. The BW was set at 70 kg (https://www.donneesmondiales.com/taille-moyenne.php).

#### 2.5.2 Non-carcinogenic risk

The estimation of the level of non-carcinogenic risk associated with the consumption of the fish was determined using the targeted hazard quotient (THQ). The threshold value for THQ is 1 [20], and higher THQ values increase the probability of experiencing long-term non-carcinogenic effects. If THQ values *<*1, toxic effects are not expected to occur [21], and in the case of THQ ≥ 1, there is a potential health hazard [22].

**THQ = MC × DR × EF × ED × 10**^**−3**^**/ BW × Atn × RFD**

Where MC is the average concentration of HMs in fish (mg.kg^−1^ dw), DR is the estimated daily consumption rate of fish (g/person/day), set at 82 g/person/day (that is 30 kg/person/year in Gabon) [5], EF is the exposure frequency to HMs (365 days a year), ED is the exposure duration to HMs (the mean life expectancy of a person was conservatively assumed to be 70 years), BW is the average body weight (70 kg) for adult people in Gabon, ATn is the mean exposure period for non-carcinogens (365 days per year × exposure number per year), RFD (mg.kg^−1^.day^−1^) is the oral reference dose estimated for daily exposure to a chemical by human populations (including the sensitive subgroups) that are likely to be without an appreciable risk of the harmful effects during a lifetime indicated by the Integrated Risk Information System (for Al: 30; As: 0.0003; Cd: 0.0005; Fe: 0.7; Hg: 0.0004; Mn: 0.024; Pb: 0.0035 mg.kg^−1^ day^−1^) [23], [24].

The hazard index (HI) for each fish species was assessed to determine the risk of all the HMs in the fish samples. The HI for each fish species is the sum of the Targeted Hazard Quotients for Al, As, Cd, Fe, Hg, Mn and Pb intakes.

**HI = ∑ THQs**

HI value *<* 1 shows that the consumption of fish is safe, and HI value *>* 1 indicates harmful effects to humans.

#### 2.5.3 Potential carcinogenic risks

In order to evaluate the probability of developing cancer over the lifetime of an individual through the consumption of contaminated fish [25], the cancer risks (CR) associated with HMs were estimated using the following equation [26]:

**CR = MC × EF × ED × DR × CSF × 10**^**−3**^**/ BW × Atc**

Where, MC is the average concentration of HMs in fish (mg_·_kg^−1^ dw), EF is the exposure frequency to HMs (365 days a year), ED is the exposure duration to HMs (the mean life expectancy of a person was conservatively assumed to be 70 years), DR is the estimated daily consumption rate of fish (g/person/day), set at 82 g/person/day (that is 30 kg/person/year in Gabon) [5], BW is the average body weight (70 kg) for adult people in Gabon, Atc is the mean exposure period for carcinogens (365 days per year × exposure number per year). CSF is the cancer slope factor for the oral route indicated by the Integrated Risk Information System (As: 1.5; Cd: 0.038; Pb: 0.0085 mg.kg^−1^ day^−1^) [23].

Based on USEPA [23], a CR value lower than 10^−6^ is seen as negligible, one within the 10^−6^ and 10^−4^ range is regarded as acceptable, and one higher than 10^−4^ is deemed unacceptable.

### 2.6 Statistical analysis

Data are presented as mean ± standard deviation unless otherwise indicated. A number of statistical analyses were used to examine whether species and seasonal variation are present in the measured HMs among the selected lakes. Metal concentrations measured from the *Chrysichthys nigrodigitatus*, *Polydactylus quadrifilis*, *Oreochromis niloticus* and *Clarias gariepinus* from each sampling season for the selected lakes were statistically analysed through a One-way ANOVA followed by Tukey’s post hoc multi-comparison test for parametric data and Kruskal-wallis followed by Dunn’s test for non-parametric data, using GraphPad Prism version 5.00 (GraphPad Software, San Diego, CA, USA).

## 3 Results

### 3.1 Metal concentrations in fish sampled: seasonal, spatial and flow-related variation

The concentrations of HMs expressed in mg·kg^−1^ dw in muscles of *O. niloticus, C. gariepinus, C. nigrodigitatus* and *P. quadrifilis* are presented in Table 2A and Table 2B. The concentrations of HMs varied between seasons, lakes and fish species. Of all the HMs assessed, the least detected was Cd. Of the 7 HMs analysed, Cd was only detected in muscle tissues of *C. nigrodigitatus* in the Oguemoué and Ezanga Lakes during the HF period. The highest HM concentrations were those of Al and Fe, and the lowest those of Cd and As. For the fish samples exhibiting As concentration above the detection limit during the HF period, a significantly higher concentration was recorded in *C. nigrodigitatus* caught at Ezanga Lake compared to *C. nigrodigitatus* and *P. quadrifilis* at Onangué and Oguemoué Lakes, respectively (p < 0.05, Table 2A). When considering the differences in As concentrations between fish species of the various lakes during the LF period, *P. quadrifilis* (Onangué Lake) had far more As in its muscle than other fish species from any other lakes (p < 0.05).

**Table 2A:** Levels of heavy metals (Al, As, Cd in mg.kg^−1^ dw) in fish muscles.

Lakes	Species	Al		As		Cd	
		HF	LF	HF	LF	HF	LF
Oguemoué	*Chrysichthys nigrodigitatus*	51.53±46.46c	68.5±58.5a	0.03±0.01b	BDL	0.01±0a	BDL
	*Polydactylus quadrifilis*	13.7±0.20d	11.60±1.17c	0.01±0.01b	0.05±0.02b	BDL	BDL
	*Oreochromis niloticus*	SNF	15±1 cd	SNF	BDL	SNF	BDL
Ezanga	*Chrysichthys nigrodigitatus*	5.78±0.23e	BDL	0.10±0.05a	BDL	0.05±0.02a	BDL
	*Polydactylus quadrifilis*	7.56±1.32e	BDL	BDL	BDL	BDL	BDL
	*Oreochromis niloticus*	SNF	BDL	SNF	BDL	SNF	BDL
Onangué	*Chrysichthys nigrodigitatus*	11.59±3.3e	8.28±1.42d	0.05±0.02b	BDL	BDL	BDL
	*Polydactylus quadrifilis*	79.12±57.71ab	11.19±1.81d	BDL	0.17±0.1a	BDL	BDL
	*Oreochromis niloticus*	72.36±56.03b	9.66±0.55d	BDL	0.06±0.0b	BDL	BDL
Nguenè	*Chrysichthys nigrodigitatus*	22±9d	9.13±5d	BDL	BDL	BDL	BDL
	*Polydactylus quadrifilis*	24±5d	13±6 cd	BDL	BDL	BDL	BDL
	*Oreochromis niloticus*	90±83a	BDL	BDL	BDL	BDL	BDL
Grand Poubara	*Clarias gariepinus*	53.77±50.29c	21.36±2.03c	BDL	BDL	BDL	BDL
	*Oreochromis niloticus*	32.66±13.89d	45.46±36.7b	BDL	0.05±0.02b	BDL	BDL

BDL **=** below detection limit, Al < 0.100 mg/kg; As < 0.001 mg/kg; Cd < 0.001 mg/kg

HF= high flow; LF = low flow

SNF = species not found

Within each sampling period (HF or LF), concentration values with different letters indicate statistical differences between the fish species across the lakes.

**Table 2B:** Levels of heavy metals (Fe, Hg, Mn, Pb in mg.kg^−1^ dw) in fish muscles.

Lakes	Species	Fe		Hg		Mn		Pb	
		HF	LF	HF	LF	HF	LF	HF	LF
Oguemoué	*Chrysichthys nigrodigitatus*	45.29±11.84b	31.23±1.22d	1.08±0.89c	2.06±1.79bc	0.95±0.34bc	1.21±0.23 cd	0.05±0b	0.06±0c
	*Polydactylus quadrifilis*	12±2e	26.47±3.84d	0.72±0.01 cd	1.34±0.66 cd	0.62±0.39c	0.75±0d	0.05±0b	0.12±0.06b
	*Oreochromis niloticus*	SNF	53±3bc	SNF	0.36±0.14 f	SNF	2.83±0.04b	SNF	0.06±0.01c
Ezanga	*Chrysichthys nigrodigitatus*	22±0.84de	BDL	0.81±0.57 cd	BDL	0.46±0.10c	BDL	0.08±0.04b	BDL
	*Polydactylus quadrifilis*	19±6e	BDL	0.90±0.25 cd	BDL	0.36±0.19c	BDL	BDL	BDL
	*Oreochromis niloticus*	SNF	BDL	SNF	BDL	SNF	BDL	SNF	BDL
Onangué	*Chrysichthys nigrodigitatus*	5.32±1.28 f	48.50±13.50c	2.54±1.11a	0.77±0.46ef	0.55±0.06c	1.20±0.52 cd	0.15±0.04a	0.08±0.00c
	*Polydactylus quadrifilis*	5.92±1.73 f	22.77±4.20d	0.67±0.20d	1.03±0.68de	0.60±0.43c	0.64±0.12d	BDL	0.06±0.00c
	*Oreochromis niloticus*	31.50±6.50 cd	70.27±13.16ab	BDL	0.17±0.03 g	2.67±0.29a	2.58±1.33b	0.05±0.00b	0.07±0.00c
Nguenè	*Chrysichthys nigrodigitatus*	41.50±19.50bc	39.12±0.12 cd	0.26±0.04e	1.49±0.08 cd	1.27±0.69b	1.50±0.11c	0.06±0.00b	0.06±0.00c
	*Polydactylus quadrifilis*	54.50±22.50ab	25.50±0.50d	1.24±0.14bc	3.52±0.02b	0.97±0.06bc	0.56±0.06d	0.07±0.00b	BDL
	*Oreochromis niloticus*	1.83±1.39 g	BDL	0.28±0.04e	BDL	0.92±0.45bc	BDL	BDL	BDL
Grand Poubara	*Clarias gariepinus*	66±16a	82±28.38a	1.67±1.19b	6.65±1.94a	1.45±0.71b	1.66±0.49c	0.05±0.00b	0.07±0.03c
	*Oreochromis niloticus*	52±1ab	60±18.29bc	0.43±0.28de	0.12±0.00 g	2.46±0.88a	6±5.33a	0.06±0.00b	0.21±0.10a

BDL= below detection limit; Fe < 0.025; Hg < 0.001; Mn < 0.025; Pb < 0.001

HF= high flow; LF = low flow

SNF = species not found

Within each sampling period (HF or LF), concentration values with different letters indicate statistical differences between the fish species across the lakes.

**Fig. 2:**
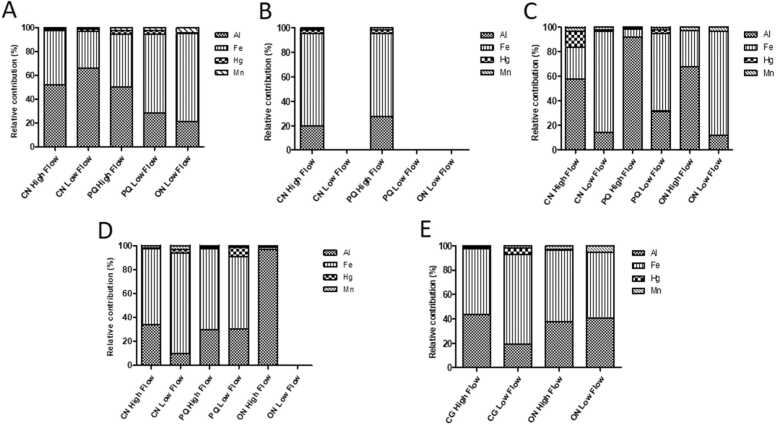
The relative contribution of the major individual metals in fish species contamination over seasonal sampling for A) Oguemoué, B) Ezanga C) Onangué, D) Nguenè and E) Grand Poubara. CN = *Chrysichthys nigrodigitatus*; PQ = *Polydactylus quadrifilis*; ON = *Oreochromis niloticus*; CG= *Clarias gariepinus.*

In all the fish muscles sampled during HF and LF periods, Al concentrations were highest in *O. niloticus* (90 mg.kg^-1^, Nguenè Lake) and *C. nigrodigitatus* (68.5 mg.kg^-1^ Oguemoué Lake), respectively (Table 2A). This was followed by Al concentrations in *P. quadrifilis* (HF, Onangué Lake) and *O. niloticus* (HF, Onangué Lake and LF Grand Poubara Lake). In the Oguemoué Lake, Al had significantly higher concentrations in *C. nigrodigitatus* than any other fish species irrespective of the seasons, while in the Grand Poubara Lake, *C. gariepinus* and *O. niloticus* exhibited higher Al concentrations during HF and LF periods, respectively. This metal was the first contributor to the HMs bioaccumulation in *O. niloticus* and *P. quadrifilis* captured during the high flow period at the Nguenè and Onangué Lakes respectively, and *C. nigrodigitatus* caught during low flow period at the Oguemoué Lake (Table 2A). Its proportions in bioaccumulated HMs in the muscles of these species were 66.46 % in *C. nigrodigitatus* at the Oguemoué Lake, 67.88 % in *O. niloticus* at the Onangué Lake, 91.67 % in *P. quadrifilis* at the Onangué Lake and 96.74 % in *O. niloticus* at the Nguenè Lake (Fig. 2A, 2C and 2D). Nevertheless, Al was below the detectable limit in the muscle tissues of all fish species found in the Ezanga Lake, and in *O. niloticus* sampled from the Nguenè Lake (Table 2A).

Throughout the seasons, and regardless of the lake and fish species, Fe was found to be the most represented metal. Compared to all the other metals, amongst the lakes studied, statistical analyses revealed that Fe concentrations varied between the fish species; during the HF period significantly higher concentrations were recorded in *C. gariepinus* and *O. niloticus* (Grand Poubara Lake) and *P. quadrifilis* (Nguenè Lake) than any other fish species, while during the LF period higher concentrations were found in *C. gariepinus* at Grand Poubara Lake and *O. niloticus* at Onangué Lake (p *<* 0.05, Table 2B). This metal was the most abundant in all these fish species and accounted for 54 % *C. gariepinus* (HF, Grand Poubara Lake), 59.34 % *O. niloticus* (HF, Grand Poubara Lake), 67.45 % *P. quadrifilis* (HF, Nguenè Lake), 73.39 % *C. gariepinus* (LF, Grand Poubara Lake) and 85 % *O. niloticus* (LF, Onangué Lake) of the HMs in fish muscle tissues (Fig. 2C, 2D and 2E). The concentration of Fe was below the detectable limit in the muscle tissues of all fish species captured during the LF period at the Ezanga Lake.

The highest and lowest concentrations of Hg were recorded in the muscle tissues of *C. gariepinus* (6.65 mg. kg^−1^) and *O. niloticus* (0.12 mg. kg^−1^) sampled during LF period at Grand Poubara Lake (Table 2B). The mean Hg concentrations increased significantly in *C. nigrodigitatus* compared to *O. niloticus* sampled during the LF period at the Oguemoué and Onangué Lakes (p *<* 0.05). During the HF period, *C. nigrodigitatus* showed more Hg concentrations than *P. quadrifilis* at Onangué Lake, while the opposite trend was observed in *P. quadrifilis* exhibiting more Hg concentration as compared to *C. nigrodigitatus* and *O. niloticus* at Nguène Lake. In *C. gariepinus* sampled during the HF period at Grand Poubara, the Hg concentration was significantly higher compared to *O. niloticus* (p *<* 0.05). At Grand Poubara during the LF period, Hg represented the third most abundant pollutant after Fe and Al (Table 2B, Fig. 2E).

The measured Mn concentrations varied between the fish species, seasons and the lakes studied. At Onangué Lake, the Mn concentration in the *O. niloticus* species caught during the LF period was significantly higher than the Mn concentrations in *C. nigrodigitatus* and *P. quadrifilis.* The trend of Mn concentration in *O. niloticus* obtained in the Grand Poubara Lake was the same as in Onangué and was significantly higher than in muscle of *C. gariepinus* (p *<* 0.05). Moreover, the Mn concentration in O. niloticus was the highest (6 mg. kg^-^^1^) recorded throughout the seasons and lakes, and contributed to 5.39 % of all bioaccumulated metals in specimens sampled at the Grand Poubara Lake during the LF period (Table 2B, Fig. 2E). In other lakes, such Oguemoué and Onangué, when the species was found during the LF period its Mn concentrations were more than in any other fish species (p < 0.05).

The Pb concentrations were similar in all the fish caught during the HF period, except in *C. nigrodigitatus* at the Onangué Lake where significantly higher Pb level was found (p < 0.05). During the LF period, Pb concentrations seemed higher in *P. quadrifilis* as compared to *C. nigrodigitatus* and *O. niloticus* at the Oguemoué Lake, while, *O. niloticus* showed far higher Pb concentration than *C. gariepinus* at the Grand Poubara Lake. Pb contribution to heavy metal bioaccumulation ranged from 0.03 % in *C. gariepinus* (LF period) at Grand Poubara to 0.74 % in *C. nigrodigitatus* (HF period) at Onangué (Fig. 2).

### 3.2 Human health risk assessment

#### 3.2.1 Estimated daily intake (DI)

The estimated daily intake (mg. kg^−1^. day^−1^) of the seven heavy metals, calculated for the four fish species is reported in Table 3. The daily intake of respective heavy metals depends on the concentration of each metal in the muscle of the different fish species and on the amount of fish consumed. The reference doses (RFD) used for the calculation are those of the American Environmental Protection Agency [23] and the tolerance dose intake (TDI) from the study by Varol et al. [24]. Table 3 shows that in all the fish species, the DI values for Al and Fe were higher than those of the other metals, followed by those of Hg and Mn. In general, the DI values of this study were lower than the RFD and TDI values, except for Hg. Fish of the species *O. niloticus* from lakes Onangué, Nguenè and Grand Poubara had DI values for Hg lower than the RFD, while all other species in the various lakes exceeded the critical consumption threshold for this metal.

**Table 3:** Estimated daily intake (DI) of heavy metals in muscle of fish species of Moyen-Ogooué and Haut-Ogooué Provinces of Gabon.

Lakes	Species	DI (mg.kg^−1^ day^−1^)						
		Al	As	Cd	Fe	Hg	Mn	Pb
Oguemoué	*C. nigrodigitatus*	7.00E−02	3.64E−05	1.55E−05	4.48E−02	1.84E−03	1.27E−03	6.76E−05
	*P. quadrifilis*	1.48E−02	2.72E−05	3.08E−06	2.25E−02	1.21E−03	8.03E−04	6.52E−05
	*O. niloticus*	1.76E−02	BD	BD	6.21E−02	4.24E−04	3.31E−03	7.96E−05
Ezanga	*C. nigrodigitatus*	6.77E−03	1.21E−04	6.03E−05	2.61E−02	9.45E−04	5.40E−04	9.78E−05
	*P. quadrifilis*	8.85E−03	BDL	BDL	2.22E−02	1.05E−03	2.14E−04	BDL
	*O. niloticus*	BDL	BDL	BDL	BDL	BDL	BDL	BDL
Onangué	*C. nigrodigitatus*	1.16E−02	6.91E−05	BDL	3.15E−02	1.93E−03	1.03E−03	1.40E−04
	*P. quadrifilis*	5.28E−02	2.03E−04	BDL	1.68E−02	9.96E−04	7.31E−04	7.10E−05
	*O. niloticus*	4.80E−02	7.11E−05	BDL	5.96E−02	1.95E−04	3.08E−03	7.65E−05
Nguenè	*C. nigrodigitatus*	1.55E−02	BDL	BDL	4.72E−02	1.03E−03	1.63E−03	7.82E−05
	*P. quadrifilis*	2.15E−02	BDL	BDL	4.68E−02	2.79E−03	9.04E−04	8.83E−05
	*O. niloticus*	1.05E−01	BDL	BDL	2.14E−03	3.33E−04	1.09E−03	BDL
Grand Poubara	*C. gariepinus*	4.40E−02	BDL	BDL	8.69E−02	4.88E−03	1.83E−03	5.29E−05
	*O. niloticus*	4.57E−02	5.88E−05	BDL	6.54E−02	3.27E−04	4.96E−03	9.95E−05

#### 3.2.2 Targeted hazard quotient (THQ), Hazard index (HI), Carcinogenic risk (CR) and Metal pollution index (MPI)

Human health risk index values (MPI, THQ, HI and CR) of the fish species for adult consumers are shown in Table 4. The metal pollution index (MPI) values ranged from 0.37 to 2.61. In general, the MPI values calculated from *C. nigrodigitatus* samples were lower than those of other fish species, except from the samples from the Nguenè Lake where the MPI value was 1.75. The highest MPI value was recorded in the *C. gariepinus* samples from the Grand Poubara Lake. Values of MPI in the muscle of *P. quadrifilis* were 0.37 mg·kg^−1^ at the Oguemoué Lake, 1.20 mg·kg^−1^ at the Onangué Lake, 1.73 mg·kg^−1^ at the Ezanga Lake and 1.93 mg·kg^−1^ at the Nguenè Lake. Values of MPI in *O. niloticus* were 1.21 mg·kg^−1^ at the Onangué Lake, 1.51 mg·kg^−1^ at the Grand Poubara Lake, 1.71 mg·kg^−1^ at the Nguenè Lake and 1.77 mg·kg^−1^ at the Oguemoué Lake.

**Table 4:** Target hazard quotient (THQ), hazard index (HI), target cancer risk (CR) and metal pollution index (MPI) of fish species following metal concentrations.

Lakes	Species	THQ							HI	CR			MPI
		Al	As	Cd	Fe	Hg	Mn	Pb		As	Cd	Pb	
Oguemoué	*C. nigrodigitatus*	8.55E+02	4.42E+04	1.13E+04	2.33E+04	1.67E+06	1.92E+04	7.05E+03	1.78E+06	19.93	0.21	0.21	0.71
	*P. quadrifilis*	1.80E+02	3.31E+04	2.25E+03	1.17E+04	1.10E+06	1.22E+04	6.79E+03	1.17E+06	14.91	0.04	0.20	0.37
	*O. niloticus*	2.13E+02	BDL	BDL	3.23E+04	3.87E+05	1.21E+03	8.30E+03	4.29E+05	BDL	BDL	0.24	1.77
Ezanga	*C. nigrodigitatus*	82.41	1.47E+05	4.39E+04	1.36E+04	8.62E+05	8.21E+03	1.02E+04	1.08E+06	66.59	0.83	0.30	0.58
	*P. quadrifilis*	1.07E+02	BDL	BDL	1.16E+04	9.62E+05	6.51E+03	BDL	9.80E+05	BDL	BDL	BDL	1.73
	*O. niloticus*	BDL	BDL	BDL	BDL	BDL	BDL	BDL	BDL	BDL	BDL	BDL	
Onangué	*C. nigrodigitatus*	1.41E+02	2.10E+04	BDL	1.64E+04	1.76E+05	1.57E+04	1.45E+04	2.44E+05	9.46	BDL	0.43	1.15
	*P. quadrifilis*	6.43E+02	1.23E+05	BDL	8.76E+03	9.09E+05	1.11E+04	3.69E+03	1.06E+06	55.68	BDL	0.11	1.20
	*O. niloticus*	5.84E+02	4.32E+04	BDL	3.11E+04	8.91E+04	4.68E+04	7.97E+03	2.19E+05	19.46	BDL	0.23	1.21
Nguenè	*C. nigrodigitatus*	1.89E+02	BDL	BDL	2.46E+04	9.41E+05	2.47E+04	8.15E+03	9.99E+05	BDL	BDL	0.24	1.75
	*P. quadrifilis*	2.62E+02	BDL	BDL	2.44E+04	2.54E+06	1.37E+04	9.21E+03	2.59E+06	BDL	BDL	0.27	1.93
	*O. niloticus*	1.28E+03	BDL	BDL	1.11E+03	3.03E+05	1.65E+04	BD	3.22E+05	BDL	BDL	BDL	1.71
Grand Poubara	*C. gariepinus*	5.35E+02	BDL	BDL	4.53E+04	4.45E+06	2.77E+04	5.51E+03	4.53E+06	BDL	BDL	0.16	2.61
	*O. niloticus*	5.56E+02	7.15E+04	BDL	3.41E+04	2.97E+05	7.54E+04	1.03E+04	4.89E+05	1.34E+05	BDL	0.30	1.51

The targeted hazard quotient (THQ) values of all heavy metals were all higher than 1 (Table 4), which showed a potentially high risk of the consumer developing cancer during his/her lifetime. This was confirmed by the HI values of each fish species that determine the potential risk of multiple metal contaminations. Carcinogenic risk (CR) values, calculated for As, Cd and Pb were all higher than the unacceptable threshold of 10^−4^.

## 4 Discussion

In the Moyen-Ogooué and Haut-Ogooué Provinces of Gabon, we assessed metal bioaccumulation in fish species from selected rivers during different flow periods and evaluated the potential risks posed to the health of local populations. Bioaccumulation studies using higher organisms of the food chain can help provide information at threefold; (i) contaminant-specific bioavailability, (ii) possible causative agents of toxicity and (iii) the link between body loads and accumulation values in the food chain [27], [28]. In bioaccumulation monitoring studies, metal residue levels in tissues of aquatic organisms have been used to draw conclusions on the potential toxicity of the present toxicants [8], [11], [29].

### 4.1 Metal bioaccumulation as an indicator of aquatic pollution

The temporal HM bioaccumulation patterns in this study may reflect their sourcing and bioavailability in the aquatic environment. The recorded HM concentrations varied between the various seasons (and water flow) to the lakes studied and HM concentrations were in general higher in the Grand Poubara Dam ˃ Onangué Lake ˃ Oguemoué Lake ˃ Nguenè Lake ˃ Ezanga Lake, although according to the seasons there were significantly higher concentrations in certain HMs in the least contaminated lakes. General trends in toxic HMs (Al, As, Cd, Hg, Pb) exclusively were as follows: Onangué Lake ˃ Grand Poubara Dam ˃ Oguemoué Lake ˃ Nguenè Lake ˃ Ezanga. This may be indicative of the preponderant anthropogenic activities occurring in and around the lakes. Moreover, HM concentrations were significantly different among fish species from lakes Onangué, Oguemoué and Ezanga, which are relatively close to one another. For the Oguemoué and Ezanga Lakes, HMs accumulation seemed higher in *C. nigrodigitatus* followed by *P. quadrifilis* and *O. niloticus*, whereas in the Onangué Lake HM accumulation tended to be higher in *P. quadrifilis*, *O. niloticus* and *C. nigrodigitatus* (Table 2A, Table 2B)*.* Differences in HM concentrations among fish species have been reported throughout the literature. Afifi et al. [30] recorded higher values of Fe, As and Pb in muscle tissues of *O. niloticus* than *C. gariepinus* caught in the El-Abbasa fish farm, Egypt. In another study by Abbas et al. [31], more Fe, Pb, Cd, Mn were found in *O. niloticus* than *Chelon ramada* farmed in aquaculture sites across El‑Sharkia and Kafr El‑Sheikh Governorates in Egypt. The literature reports varying bioaccumulation factors of HMs in fish muscles depending on endogenous factors such the fish eating behaviour, physiological state, age, etc., and exogenous factors such as metal bioavailability, water physicochemical properties, etc. [32]. In the current study, the age of the fish was not assessed for HM contamination. This could have provided relevant information on their probable time exposure to the aquatic environment they lived in. At Grand Poubara Lake, in the area of Franceville, *C. gariepinus* seemed to show some of the highest concentrations of Hg ever reported on the African continent, especially during the LF period. In a study by Abbas et al. [33] conducted on *C. gariepinus* bred in fish farm and those from the wild in Borollus Lake in Egypt, similar Cd (0.03–0.05 vs. 0.01–0.05 mg.kg^−1^) and Fe (12.55–13.94 vs. 10.30–11.69 mg.kg^−1^) concentrations, but higher As (0.23–1.08 vs. 0.01–0.05 mg.kg^−1^) and Pb (0.05–0.21 vs. 1.07–2.20 mg.kg^−1^) concentrations were recorded in wild samples than bred samples. This, suggested that the sources of contamination may come from either the core of the lakes or the different run-off profiles of each lake. When considering the differences in the recorded HM concentrations between the LF and HF periods, the majority of HMs had higher concentrations during the HF period in all the lakes, suggesting a rise of metal contamination in general in rainy season characterized by relatively low temperature compared to dry season. Iordache et al. [34] attributed the increase in metal levels and implicitly fish biomass from Romanian freshwaters to low water flow and high temperatures. However, increased concentrations of HMs in fish from the studied lakes during HF period may be attributed to additional inputs of HMs. Rainfall events and increased runoff can lead to the erosion and transport of HMs from terrestrial sources into aquatic ecosystems [34], and since they are not biodegradable they end up bioaccumulating in the water environment [35]. The lands around the studied lakes are used for agricultural activities, and as in tropical agriculture application of agrochemicals tends to increase during the rainy season [36].

Heavy metal contamination in studied lakes follows the decreasing order Fe > Al > Mn > Hg > Pb > As > Cd. The Fe concentrations in muscle tissues of *O. niloticus* (LF) at Oguemoué, *C. nigrodigitatus* (LF) and *O. niloticus* (LF) at the Onangué, *P. quadrifilis* (HF) at the Nguenè, *C. gariepinus* (LF and HF) and *O. niloticus* (LF and HF) at Grand Poubara, were far above those found by Reda and Ayu [37] for *O. niloticus* and *C. gariepinus* species in the Chamo Lake, Ethiopia (18.98–40.20 mg.kg^−1^ dw). In almost all the lakes and fish species studied, the Fe concentrations far exceeded those reported for *O. niloticus* (0.80 mg.kg^−1^) and *C. gariepinus* (18.01 mg.kg^−1^) species in Keban Lake, Turkey [38]. However, the Fe measured in fish species from the present study, were below the threshold value for Fe (100 mg.kg^−1^) recommended by the World Health Organization [39]. Higher concentrations of Mn than recommended by international standards were found in *C. nigrodigitatus, O. niloticus* and *C. gariepinus* during either HF or LF periods at Oguemoué, Onangué and Nguenè (Table 2B). Some Mn concentrations were slightly higher than those obtained in fish of the same species in Borullus (0.23 mg.kg^−1^) and Edku Lakes (1.98 mg.kg^−1^), but lower than those recorded at Manzala Lake (22.98 mg.kg^−1^) in Egypt by Saeed and Shaker [40]. According to the World Health Organization, fish muscle’s maximum allowable Mn content is 1 mg.kg^−1^ of the body weight [39].

Aluminium concentrations in fish species from all the lakes during both LF and HF periods were far above the recommended values of 0.10 and 0.12 mg.kg^−1^ [41]. The high levels and abnormal accumulation of Al in all the fish species can be attributed to the acidification of lakes through industrial discharge and effluent from wastewater treatment plants [42]. Mercury is one of the most toxic metals in the studied lakes, as seen by the high concentrations found in certain fish species. The concentration of Hg found in *C. gariepinus* during the LF period at Grand Poubara was nine to forty times greater than those found in Nyeri County in Kenya (0.157 mg.kg^−1^ [43]) and Asaba Markets in Nigeria (0.723 mg.kg^−1^ [44]), respectively. Manz Koule et al. [45] reported Hg concentration of 0.8 mg.kg^−1^ in *P. quadrifilis* in seaport of Douala in Cameroon. Dry mass concentrations of Hg recorded in the present study were, in most fish species (and lakes) higher than the maximum allowed concentrations of Hg in fish (˂0.5 mg.kg^−1^) imposed by the European Commission [46], the Food and Agriculture Organization [47], [48]. This clearly indicates the high levels of exposure of water courses of the area to mercury, which may be sourced from clearing activities and deforestation occurring in the Moyen-Ogooué and Haut-Ogooué Provinces of Gabon.

Arsenic and cadmium concentrations were the lowest of all the HMs measured. Arsenic concentrations recorded were similar to the As levels reported in fish species from the Tigris River, Turkey (0.02–0.20 mg.kg^−1^), but far below those of the Danube River, Serbia (7.72 mg.kg^−1^), and the Ogun River, Nigeria (35–130 mg.kg^−1^) [8], [11], [49]. Generally, Pb concentrations across the studied lakes were below the recommended threshold by the Food and Agriculture Organization [47], the European Commission [46], Food Standards Australia New Zealand [50] and the Chinese Ministry of Health [51], which are 0.2, 0.3, 0.5 and 0.5 mg.kg^−1^, respectively. Recently, Lazǎr et al. [29] reported a negative correlation between water level and the concentrations of Pb in the tissues of the Pontic shad (*Alosa immaculate*) from the Danube River. The same trend was observed in the present study. Differences in HM levels between fish species may exhibit various sources of contamination of lakes by HMs depending upon spatial location, natural geological origin and anthropogenic activities occurring in the area. Human activities such as agriculture, waste incineration, fossil fuel combustion, petrochemical activities, coupled with natural sources of contamination (soil erosion, leaching, forest fires) are the main sources of HMs in the aquatic environment [52].

In the present study, observed changes in fish population dynamics deserve to be reported. For example, the absence of the Nile tilapia (*O. niloticus*) during the HF period in the Oguemoué and Ezanga Lakes was observed. This may be due to the tendency of this species to migrate to a more favourable environment (typified by relatively lower water levels). Since the Oguemoué, Ezanga and Onangué lakes are linked via channels, it may have migrated to the Onangué Lake or other small lakes around as a result of the increased volume of water in the Oguemoué and Ezanga Lakes.

### 4.2 Human health risk assessment

Certain HMs may enter the human body through the accumulation in the food chain, where they either interfere with enzyme activity by forming bonds with sulfur groups in enzymes, or attach to cell membranes and obstruct transport mechanisms across cell walls, causing harm to human health [53], [54]. In the present study, the risk of ingested fish meat by adults revealed that, except for Hg, HM concentrations did not exceed the acceptable daily intakes [23]. Approximately 78 % of the fish samples exceeded the acceptable limits for Hg set by the United States Environmental Protection Agency (0.0004 mg·kg^−1^.day^−1^, [23]). The average percentage differences between the limits established by USEPA [23] and the levels of Hg ranged from 99.91 % (LF) to 99.93 % (HF). The probable explanation behind the exceeded limit concentrations of Hg, may be attributed to its capacity to be methylated by anaerobic bacteria present in water into methylmercury, which is a highly bioaccumulative (and toxic) form of organic mercury that biomagnifies in fish species [6]. Therefore, the frequent consumption of these fish species could present a health risk, thus hazard index (HI) values demonstrated potential harmful effects to humans consuming all the fish species examined. Harmful implications of Hg to human systems include among others cardiovascular (causing high blood pressure, myocardial infarction and cardiomyopathy), immunological (causing either immuno-stimulant, immuno-suppresant, or lympho-proliferant), Nephrological (causing kidney dysfunction), reproductive (causing reduction of sperm count and motility, impaired fertility and pregnancy), neurological (causing visual dysfunction, headaches, memory loss, depression, epilepsy and cancer), respiratory (causing pneumonitis and overt respiratory failure), genetic and molecular systems (microtubular disruption, increase of intracellular Ca^2+^ and alterations of neurotransmitter function) [55], [56], [57].

The assessment of health risks associated with the consumption of the fish species further indicated an increased cancer risk in local populations consuming these fish daily over an average lifetime of 70 years. Our results are in agreement with those of Adegbola et al. [8] who reported similar cancer risks for As, Cd and Ni for consuming fish species from the Ogun and Eleyele Rivers in Nigeria. Certain HMs such as Fe and Mn are considered essential to biological organisms, while others like As, Cd, Al, Hg and Pb are non-essential HMs [7], [29]. In the human body, Cd and Pb affect, among others, kidneys, bones, lungs and DNA, and Pb particularly can cause nephropathy and kidney failure [58], [59]. The toxicity of As varies depending on the chemical speciation, with the As^3+^ species being more toxic than any other forms [60]. The presence of As species in food has been shown to cause many types of cancer, neurological, dermatitis, and cardiovascular diseases [61].

## 5 Conclusions

Monitoring the bioaccumulation of HMs in higher organisms of the food chain is crucial to understanding the evolution of contamination and related risks to aquatic ecosystems and human health. This is the first comprehensive reporting of HMs contaminant in fish species of the Moyen-Ogooué and Haut-Ogooué Provinces in Gabon. Our results showed that all the fish species accumulate HMs differently and that the amount of HMs in muscle tissues depends upon seasonal water flow levels. Alarmingly, high concentrations of toxic HMs such Hg was found in all fish species from all the lakes, which may have serious human health and ecological consequences. Consequently, an increased surveillance programme at the national level involving authorities from fishing services, public health, environmental protection, and conservation organisations should be implemented. This will help raise awareness and improve the management of fishery resources while preserving the environment and the health of local populations that rely upon these resources for their subsistence.

## CRediT authorship contribution statement

**Michel Mathurin Kamdem:** Writing – review & editing, Visualization, Formal analysis, Data curation, Conceptualization. **Patricks Voua Otomo:** Writing – review & editing, Supervision, Resources, Funding acquisition. **Gaël Darren Maganga:** Writing – review & editing, Visualization, Validation, Supervision, Resources, Methodology, Funding acquisition, Conceptualization. **Clency Okouyi Mikala:** Writing – original draft, Methodology, Formal analysis, Conceptualization.

## Declaration of Competing Interest

The authors declare that they have no known competing financial interests or personal relationships that could have appeared to influence the work reported in this paper.

## Data Availability

Data will be made available on request.
